# Scanning Electron Microscopic Evaluation of the Internal Fit Accuracy of 3D-Printed Biphasic Calcium Phosphate Block: An Ex Vivo Pilot Study

**DOI:** 10.3390/ma14061557

**Published:** 2021-03-22

**Authors:** Su-Hee Jeon, Young Woo Song, Jae-Kook Cha, Jeong-Won Paik, Sang-Sun Han, Seong-Ho Choi

**Affiliations:** 1 Department of Periodontology, Research Institute for Periodontal Regeneration, Yonsei University College of Dentistry, Seoul 03722, Korea; ssuhee929@yuhs.ac (S.-H.J.); tigger09@yuhs.ac (Y.W.S.); chajaekook@yuhs.ac (J.-K.C.); jpaik@yuhs.ac (J.-W.P.); 2 Department of Oral and Maxillofacial Radiology, Yonsei University College of Dentistry, Seoul 03722, Korea; sshan@yuhs.ac

**Keywords:** ex vivo study, computer aided design, 3D-printing, 3D-milling, scanning electron microscope

## Abstract

The aim of this study was to assess the internal fit accuracy of a three-dimensional (3D)-printed biphasic calcium phosphate (BCP) block compared with a 3D-milled poly methyl methacrylate (PMMA) block by scanning electron microscope (SEM) analysis. In a total of 20 porcine rib bones, two different types of defects having two adjacent walls and a floor were produced: a defect with a flat floor (flat defect; *N* = 10) and a defect with a concave floor (curved defect; *N* = 10). Each defect was grafted with either the 3D-printed BCP block or the 3D-milled PMMA block fabricated following the computer aided design. The defects were then cut cross-sectionally and evaluated under the SEM. The extents of internal contact and gap were measured and statistically analyzed (*p* < 0.05). All blocks in both BCP and PMMA groups were successfully fit to the flat and curved defects. The internal contact ratio was significantly higher in the BCP group (flat defect: 0.47 ± 0.10; curved defect: 0.29 ± 0.05) compared with the PMMA group (flat defect: 0.21 ± 0.13; curved defect: 0.17 ± 0.04; *p* < 0.05). The internal gap area was similar between the two groups regardless of the defect types (*p* > 0.05). The internal fit accuracy of the 3D-printed BCP block was reliable in both the flat and curved defects when compared with the accuracy of the 3D-milled PMMA block.

## 1. Introduction

Since placing implants in an augmented site was found to produce long-term reliable outcomes in several studies, guided bone regeneration (GBR) has become a routine procedure in clinics [1]. Among various types of bone substitutes and barrier membranes, a combination of particulate-type xenograft and resorbable collagen membrane is most commonly applied as it is known to be more convenient and has lower risk of postoperative complications [2]. Nonetheless, particulate bone substitute has weaknesses in terms of positional stability of the graft material and space maintenance, both of which are prerequisites for successful bone regeneration [3,4].

Attempts to overcome these disadvantages have involved using pins to secure the particulate bone substitutes underneath the barrier membrane or applying screws to fix the block bone grafts [5]. When using block bone substitutes, it is necessary to manually shape and adapt the material exactly into the recipient sites. Otherwise, severe complications, such as graft material exposure, wound dehiscence, or postsurgical infection, may occur due to insufficient fixation of the bone blocks, which consequently impedes new bone formation.

With the advances in computer aided design and computer aided manufacturing (CAD/CAM) technologies, it became possible to manufacture the three-dimensional (3D)-printed bone substitutes, which can be customized to the individual defects [6]. Based on cone-beam computed tomography (CBCT), surgeons can virtually design and fabricate a customized graft material by analyzing the size and shape of the patients’ bone defect [7]. It is more accurate and convenient to use customized bone grafts as they do not require additional effort for the manual shaping procedure during surgery but increase the positional stability, which can eventually relieve the discomfort of both the clinicians and patients.

One of the most-used materials for producing synthetic bone block is biphasic calcium phosphate (BCP), which consists of hydroxyapatite (HA) and β-tricalcium phosphate (TCP). Previous studies reported the clinical efficacy of using 3D-printed synthetic bone graft materials in bone regeneration [8]. The accuracy of the block-type bone substitute is a key factor for stability and is crucial for a successful GBR. A few studies evaluated the extent of the adaptation of the 3D-printed bone grafts; however, most of the studies used relatively subjective methods [9]. No standard has been proposed for evaluating the suitability of the customized graft materials. The internal adaptation of resin-based (i.e., poly methyl methacrylate (PMMA)) prostheses, fabricated by CAD/CAM and the milling technique, produces consistent and reliable performance compared with the conventionally made ones [10,11]. It could be suitable to compare the internal fit accuracy of the 3D-printed BCP with the 3D-milled PMMA. The aim of the present study was to evaluate the internal fit accuracy of the 3D-printed BCP block compared with the 3D-milled PMMA block by scanning electron microscope (SEM) analysis.

## 2. Materials and Methods

### 2.1. Study Design

The present pilot research was conducted as an ex vivo experiment using porcine rib bones. Due to the nature of the study design, any kind of experimental guideline or ethical approval was not required.

### 2.2. Defect Formation

**Figure 1:**
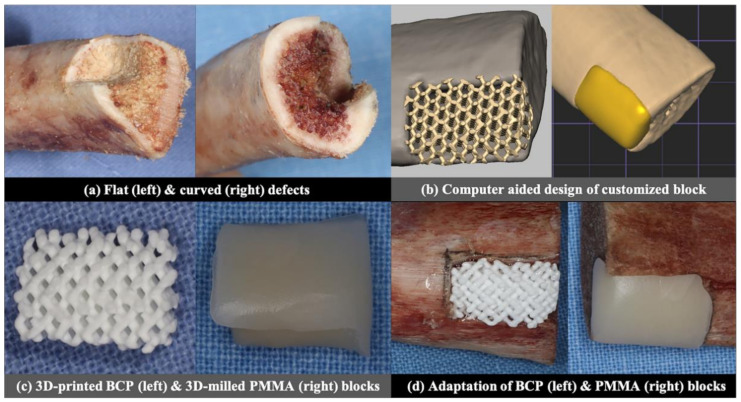
Summary of the experimental protocol.

Two different types of defects were produced in a total of 20 pig rib bones (Figure 1a): ten defects of 5 mm (width) × 5 mm (depth) × 10 mm (height) with a flat defect floor and two adjacent walls (flat defects), and the rest of the ten defects were 5 mm (width) × 5 mm (depth) × 10 mm (height) with a concave floor and two adjacent walls (curved defects). The defects were prepared manually by a single researcher (S.-H.J.) with a diamond bur and low-speed rotary engine at both edges of the rib bone with the flat defect at one side and the curved defect at the opposite side.

### 2.3. Group Allocation

Either a HA-/β-TCP block or PMMA resin block was adapted to the flat and curved defects based on the following group allocation:

BCP group: customized, 3D-printed HA-/β-TCP block;

PMMA group: customized, 3D-milled PMMA resin blocks.

### 2.4. Fabrication and Adaptation of Customized BCP and PMMA Blocks

#### 2.4.1. Virtual Design of the BCP and PMMA Blocks

CBCT was taken for both the flat and curved defects (Alphard3030, Asahi Roentgen Ind., Co. Ltd., Kyoto, Japan), under a voltage of 70 KV, current of 3 mA, voxel size of 0.30 mm, 154 mm × 154 mm FOV and 360° rotation. The images were exported as digital imaging and communication in medicine (DICOM) files. The DICOM files were converted into stereolithographic (STL) files using image processing software (Materialise Mimics 20.0, Materialise, Leuven, Belgium). Then, CAD software (3-MaticMaterialise Mimics 20.0, Materialise, Leuven, Belgium) was used to design the customized BCP and PMMA blocks, which were adapted to the defect model without a space for the cement between the surfaces of the block and the defect (Figure 1b).

#### 2.4.2. 3D Fabrication of the BCP and PMMA Blocks

The design of the BCP block was loaded into the 3D printer (CUBICON Lux DLP—B12C, Cubicon, Korea). The BCP slurry consisted of hydroxyapatite and β-tricalcium phosphate (TCP) in a weight ratio of 60: 40 (Osteon III block, Genoss, Suwon, Korea), and went through the digital light processing (DLP) by photo-curing of the biphasic calcium phosphate slurry, followed by sintering and sterilization [12] to produce a block with 1200 μm macropores. The design of the PMMA block was transferred to the milling machine (M4 Wet Heavy Metal milling unit, Zirkonzahn, Italy), which milled the raw block of PMMA (VIPI Block, Dentsply, Brazil) to make a customized PMMA block (Figure 1c).

#### 2.4.3. Adaptation of the Blocks to the Defects

The BCP and PMMA blocks were fixed to the defects with a constant force of 0.25 N, measured by a force gauge (Dial Tension Gauge, Teclock, Japan) by an adhesive agent (Histoacryl; B. Braun Surgical SA, Barcelona, Spain), which was applied over the outer boundary between the block and defect (Figure 1d).

### 2.5. Scanning Electron Microscope (SEM) Analysis

**Figure 2:**
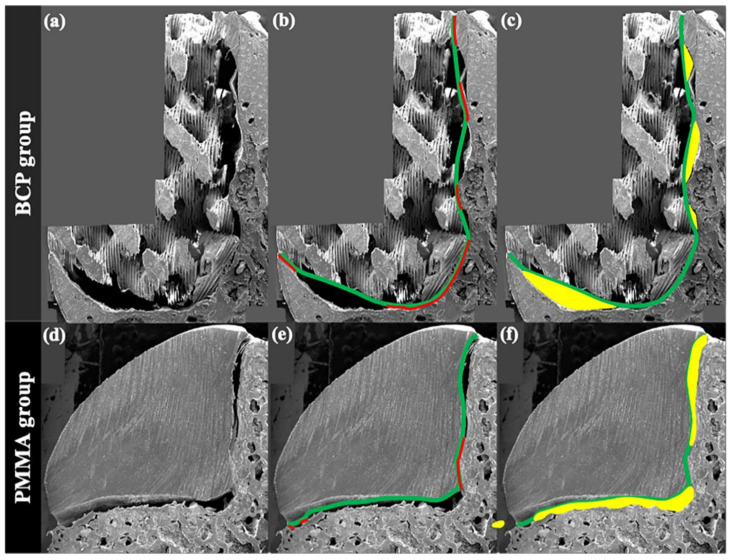
SEM analysis. (**a**) Biphasic calcium phosphate (BCP) block; (**b**) imaginary line (green) of connecting peaks of the BCP block and the line showing the actual contact with the defect (red); (**c**) internal gap area between imaginary line and defect (yellow); (**d**) poly methyl methacrylate (PMMA) block; (**e**) outline of the PMMA block (green) and the line showing the actual contact with the defect (red); (**f**) internal gap area between imaginary line and defect (yellow).

To observe the gap between the surface of the blocks and the margin of the defects in a cross-sectional view, the specimens were cut in half in the longitudinal direction and underwent a 100 nm thick platinum coating process by an ion sputter (E1010, Hitachi, Tokyo, Japan), which was followed by the observation under SEM (S-3000N, Hitachi, Tokyo, Japan) (Figure 2a,d). The obtained cross-sectional SEM images were then uploaded to computer software (Photoshop 2020, Adobe, San Jose, CA, USA), and the following parameters were measured.

#### 2.5.1. Ratio of Internal Contact between the Block and Defect

To assess the extent of internal contact, the method used for estimating the contact ratio between the dental implant and bone tissue was modified [13,14]. In previous research, the extent of direct contact between the viable bone tissue and the dental implant was represented as the ratio of actual contact length to the total length of implant surface in two-dimensional histologic images cross-sectionally cut in the middle. This method was modified in the present study as follows:

BCP group: the sum of the actual contact lengths (mm), measured between the defect and an imaginary line connecting the peaks of the BCP block outline, divided by the total length (mm) of the defect margin;

PMMA group: the sum of the actual contact lengths (mm), measured between the defect and the PMMA block surface, divided by the total length (mm) of the defect margin.

#### 2.5.2. Area of Internal Gap between the Block and Defect

For the BCP group, this parameter was measured as the sum of internal gap areas (mm^2^) between the defect and an imaginary line connecting the peaks of the BCP block outline.

For the PMMA group, it was the sum of internal gap areas (mm^2^) between the defect of the outline of the PMMA block.

The mean gap distance, which was estimated by the gap area divided by the total length not in contact with the block, was also suggested; however, it was measured in the flat defects only as the curved defects did not have a straight defect margin.

### 2.6. Statistical Analysis

Statistical analyses were carried out with computer software (IBM SPSS Statistics, Version 25.0. Armonk, NY, USA). Due to the small sample size, a nonparametric Mann–Whitney U-test was conducted for intergroup comparisons, comparing the outcome of the BCP group to that of the PMMA group within each flat and curved defect, and the outcome in the flat defect to the curved defect within each of the BCP and PMMA groups. Statistically significant was set at *p* < 0.05.

## 3. Results

### 3.1. Preliminary Evaluation of the Defect Dimension in the Cross-Section SEM Images

Both the BCP and PMMA blocks were successfully fit to the flat and curved defects without any events such as fracture or deformity during the adaptation. To verify that the dimensions of the defects were statistically similar between the BCP and PMMA groups, the total length of each flat defect and curved defect was compared between the two groups in the cross-sectional SEM images prior to the measurement of the internal contact ratio and gap area. Consequently, the total lengths of the flat and curved defects were not significantly different in both the BCP and PMMA groups (*p* > 0.05).

### 3.2. Internal Contact Ratio

In the intergroup comparisons within each type of defect, the internal contact ratios of the BCP group (flat defect: 0.47 ± 0.10; curved defect: 0.29 ± 0.05) were significantly higher than those of the PMMA group (flat defect: 0.21 ± 0.13; curved defect: 0.17 ± 0.04; *p* < 0.05).

In the intragroup comparisons between the two defects, the contact ratio measured in the flat defect was significantly higher (+0.18) than that measured in the curved defect in the BCP group (*p* < 0.05). In the PMMA group, the ratio estimated in the flat defect was also higher (+0.04) than that in the curved defects; however, the difference was not statistically significant (*p* > 0.05).

All of the measurements are summarized in Table 1.

### 3.3. Area of Internal Gap between the Block and Defect

In the flat defects, the internal gap areas were highly similar between the BCP group (0.79 ± 0.45 mm^2^) and the PMMA group (0.79 ± 0.48 mm^2^; *p* > 0.05). The mean gap distance in the flat defects was higher in the BCP group (0.14 ± 0.07 mm) compared to the PMMA group (0.08 ± 0.04 mm); however, the difference was not statistically significant (*p* > 0.05). The areas measured in the curved defects were also comparable between the two groups (BCP group: 2.49 ± 0.62 mm^2^; PMMA group: 2.23 ± 0.52 mm^2^; *p* > 0.05).

Within the BCP group, the internal gap area estimated in the curved defect were significantly larger (+1.70 mm^2^) than of that estimated in the flat defect (*p* < 0.05), which was similarly found in the comparison within the PMMA group, showing a significantly larger (+1.44 mm^2^) value in the curved defects compared with the flat defects (*p* < 0.05).

All the measurements are summarized in Table 2.

## 4. Discussion

In the present study, the extent of internal fit and discrepancy between the customized blocks (BCP and PMMA) and the defects (flat and curved) were assessed ex vivo. Overall, regardless of the defect type, the 3D-printed BCP block was more accurately fabricated than the 3D-milled PMMA block in terms of internal fit. Compared with the 3D-milled PMMA block, the 3D-printed BCP block was in contact with the defect surface: twice more in flat defects and 70.6% more in curved defects. However, the gap area between the block and the defect were comparable between the two groups. In both groups, the flat defect allowed the blocks to be fabricated more precisely, while more deviation resulted in the curved defects.

Mechanical stability achieved between the graft and the defect margin is an important factor in maintaining vascular supply, which is also related to the formation of new bone in the recipient site [15,16]. For the block bone substitute, the contact against the defect surface is considered to be closely related to the immobilization of the block. Previous studies have demonstrated that the customized graft materials and the recipient defect sites were closely matched [9,17]. However, the values that represent the extent of the fit have rarely been mentioned. Both the BCP and PMMA blocks, designed and manufactured by a digital workflow (CAD/CAM), were successfully fitted in the recipient site without additional manipulation in this study. The BCP block seemed to be reliable in terms of having a stable fit toward the defect, since the present experiment revealed that it showed significantly more contacts compared to the PMMA block. The difference in the material and the way it was used for fabricating the block might have influenced the result; however, this should be verified in the future. The results should be also interpreted with caution, as previous studies reporting the extent of the contact between the defect and the block made of either BCP or PMMA are scarce so far. Further research is needed to strengthen and establish the evidence.

Unlike the result in the internal contact, internal gap areas were similar between the two groups. Furthermore, despite the lack of statistical significance, the mean gap distance estimated in the flat defects was higher in the BCP group compared with the PMMA group. The mean gap distance of the PMMA group (0.08 ± 0.04 mm) was 20–40 μm lower than the range of 100–120 μm, which was reported clinically acceptable in the previous publications [18,19]; however, that measured in the BCP group (0.14 ± 0.07 mm) was 20–40 μm higher than the previously suggested range. This may be due to the topography of the BCP block, which had a more complicated design with 1200 μm sized macropores. These pores of the bone substitutes play a critical role in optimizing the space for the osteogenic cells and factors to occupy and the newly formed bone to reside [20,21]. Thus, it could be considered that the gap formed between the BCP block and the defect surface is not a weakness as long as the extent of the contact between the block and the defect is sufficient. So far, appropriate pore size is still controversial. Some previous studies suggested that macropores in the range of 200 to 900 μm are more effective in bone regeneration [22,23], whereas others reported that a pore smaller than 200 μm is optimal [24]. It could be assumed that a different result, such as increased contact as well as a decreased gap area, could be obtained if the pore size was reduced, and therefore, further studies should be conducted with the BCP blocks having diverse macropore dimensions.

To mimic the alveolar ridge defects in the clinical situation, two different kinds of defects, flat floor defect and curved floor defect, were prepared in a porcine rib bone. Regardless of the block type, the extents of both contact and gap were inferior in the curved defect compared with the flat defect. This finding is consistent with a previous study, which showed the increased inaccuracy of 3D fabrication when the defect margin was not even [25]. The statistical significance in the difference of the contact ratio between the two defect types appeared only in the BCP group. The reason for this outcome might be the more complex design of the BCP block compared with the PMMA block, having a relatively simple shape. Several previous publications suggested the necessity of the advance in 3D printing, as the materials have a complicated topography [26]. Nonetheless, the results should be cautiously interpreted as the internal gap areas of both groups were significantly lower in the flat defects compared with the curved defects. As most of the defects clinicians confront have uneven margins (i.e., a ridge with chronic atrophy or an extraction socket), it is necessary to enhance the performance of CAD/CAM in terms of the internal fit accuracy toward the defects with a variety of morphologies.

There are some limitations in the present research. Firstly, due to the difficulty of 3D-milling because of the BCP block having multiple macropores and the lower accuracy of the 3D printing of the dense PMMA block, the experimental material, which was directly compared, went through different fabrication modalities. This idea was based on a previous study that confirmed the similar accuracy of 3D milling and 3D printing [27]. Secondly, the assessment in the present experiment was conducted two-dimensionally. Microcomputed tomography could have allowed 3D evaluation; however, it was thought to be inappropriate owing to its insufficient resolution and definition for distinguishing the block surface and defect clearly, and so SEM was chosen instead. Thirdly, since previous studies measuring the extent of internal contact of the block-type material are scarce, the methodology used for estimating the internal contact ratio in the present experiment was modified from the method of assessing bone-to-implant contact used in the field of implant dentistry. In previous research, the fit accuracy in prostheses was measured by the gap distance estimated between the superimposed STL files. However, this was not feasible for evaluating the internal gap in the present experiment since the STL only shows the surface topography, so SEM images were used instead of STL images. This measuring method needs to be strengthened and supported by future research. Despite these limitations, this is the first experiment to evaluate the internal fit accuracy of a 3D-printed BCP block compared with other well-studied types of 3D-fabricated material, to the best of our knowledge. Further studies are needed to confirm the present outcomes in in vivo and clinical research. The present outcomes need to be confirmed in in vivo and clinical research, and the internal fit accuracy of the 3D-printed BCP blocks with various composition ratios and designs must be further evaluated.

## 5. Conclusions

Within the limitation of the study, when compared with the 3D-milled PMMA block, the 3D-printed BCP block showed a reliable internal fit accuracy in both the defects with a flat floor and curved floor. A higher extent of contact was found when the 3D-printed BCP block was applied to the flat defect compared with when applied to the curved defect.

## Figures and Tables

**Table 1:** The ratio of internal contact between the block and defect measured in the cross-sectional scanning electron microscope image.

Defect	Group	Total Length of the Defect	Contact Length between the Block and Defect	Contact Ratio
Flat	BCP	11.21 ± 0.40 mm *	5.24 ± 1.29 mm	0.47 ± 0.10 ^+,^*
	PMMA	12.41 ± 1.73 mm *	2.75 ± 2.02 mm	0.21 ± 0.13 ^+^
Curved	BCP	14.45 ± 1.04 mm *	4.14 ± 0.65 mm	0.29 ± 0.05 ^+,^*
	PMMA	16.39 ± 2.39 mm *	2.89 ± 0.93 mm	0.17 ± 0.04 ^+^

^+^ intergroup comparison within the same defect. * intragroup comparison between the two defects.

**Table 2:** The area of internal gap between the block and defect measured in the cross-sectional scanning electron microscope image.

Defect	Group	Gap Area	Mean Gap Distance
Flat	BCP	0.79 ± 0.45 mm^2^ *	0.14 ± 0.07 mm
	PMMA	0.79 ± 0.48 mm^2^ *	0.08± 0.04 mm
Curved	BCP	2.49 ± 0.62 mm^2^ *	Not measurable
	PMMA	2.23 ± 0.52 mm^2^ *	Not measurable

* intragroup comparison between the two defects.

## Data Availability

Data are contained within the article.
